# Calcium electroporation induces stress response through upregulation of HSP27, HSP70, aspartate β-hydroxylase, and CD133 in human colon cancer cells

**DOI:** 10.1186/s40659-025-00591-9

**Published:** 2025-02-21

**Authors:** Anna Szewczyk, Nina Rembiałkowska, Jolanta Saczko, Małgorzata Daczewska, Vitalij Novickij, Julita Kulbacka

**Affiliations:** 1 Department of Molecular and Cellular Biology, Faculty of Pharmacy, Wroclaw Medical University, Wrocław, Poland; 2 State Research Institute Centre for Innovative Medicine, Department of Immunology and Bioelectrochemistry, Vilnius, Lithuania; 3 Department of Animal Developmental Biology, Faculty of Biological Sciences, University of Wroclaw, Wrocław, Poland; 4 Faculty of Electronics, Vilnius Gediminas Technical University, Vilnius, Lithuania

**Keywords:** Colon cancer, Calcium ions, Electroporation, Oxidative stress, Heat shock proteins

## Abstract

**Background:**

Electroporation (EP) leverages electric pulses to permeabilize cell membranes, enabling the delivery of therapeutic agents like calcium in cancer treatment. Calcium electroporation (CaEP) induces a rapid influx of calcium ions, disrupting cellular calcium homeostasis and triggering cell death pathways. This study aims to compare the cellular responses between microsecond (µsEP) and nanosecond (nsEP) electroporation, particularly in terms of oxidative stress, immune response activation, and cancer stem cell (CSC) viability in drug-resistant (LoVo Dx) and non-resistant (LoVo) colorectal cancer cell lines.

**Results:**

Both µsEP and nsEP, particularly when combined with Ca^2+^, significantly reduced the viability of cancer cells, with nsEP showing greater efficacy. Reactive oxygen species (ROS) levels increased 5-fold in malignant cells following nsEP, correlating with decreased ATP production and mitochondrial dysfunction. Nanosecond CaEP (nsCaEP) also induced significant expression of aspartate-β-hydroxylase (ASPH), a protein linked to calcium homeostasis and tumor progression. Moreover, nsEP led to heightened expression of heat shock proteins (HSP27/70), indicating potential immune activation. Interestingly, nsEP without calcium drastically reduced the expression of CD133, a marker for CSCs, while the addition of Ca^2+^ preserved CD133 expression. The expression of death effector domain-containing DNA binding protein (DEDD), associated with apoptosis, was significantly elevated in treated cancer cells, especially in the nucleus after nsCaEP.

**Conclusions:**

The study confirms that nsEP is more effective than µsEP in disrupting cancer cell viability, enhancing oxidative stress, and triggering immune responses, likely through HSP overexpression and ROS generation. nsEP also appears to reduce CSC viability, offering a promising therapeutic approach. However, preserving CD133 expression in the presence of calcium suggests complex interactions that require further investigation. These findings highlight the potential of nsCaEP as an innovative strategy for targeting both cancer cells and CSCs, potentially improving treatment outcomes in colorectal cancer. Further studies are needed to explore the exact cell death mechanisms and optimize protocols for clinical applications.

## Introduction

Electroporation is a technique that utilizes electric pulses to temporarily permeabilize cell membranes by creating aqueous pores in the phospholipid bilayer [1–2]. The characteristics of these pores, including their size and number, depend on the specific parameters of the applied pulses, such as duration, amplitude, and frequency. The strength of the electric pulses determines whether electroporation is reversible or irreversible. Reversible electroporation can be further categorized into millisecond (msEP), microsecond (µsEP), and nanosecond (nsEP) electroporation [3], each of which has applications in various fields, including microbiology, biotechnology, medicine, and food processing [4]. Recently, electroporation has gained increasing attention in oncology as a tool for enhancing cancer treatment strategies, particularly in colorectal cancer.

One of the most promising electroporation-based therapies in oncology is electrochemotherapy (ECT), which selectively enhances drug delivery (e.g., bleomycin, cisplatin) to tumor cells [5]. Calcium electroporation (CaEP) is a novel variation of ECT that uses electric pulses to introduce extracellular calcium ions into cells. Calcium ions play a crucial role in numerous cellular processes, including gene transcription, proliferation, differentiation, and cell death [6–9]. While µsEP is a well-established anticancer treatment [10–12], the therapeutic potential of nsEP remains less explored. The key distinction between these two types of electroporation is their level of cellular penetration: µsEP primarily affects the external cell membrane, whereas nsEP can also permeabilize intracellular organelles, including the endoplasmic reticulum (ER) [13–14]. This is particularly relevant for CaEP, as nsEP-mediated calcium influx may not only increase cytosolic Ca²⁺ levels but also disrupt ER calcium stores, triggering calcium-induced calcium release (CICR) [6, 15–16]. The rapid influx of Ca^2+^ into tumor tissues destabilizes calcium homeostasis, overloads mitochondrion, and causes ATP depletion, leading to cell component distribution eventually [17–21]. Given the high metabolic activity and energy demands of colorectal cancer cells, this mechanism may be particularly relevant for their targeted eradication.

Recent studies have highlighted the significance of aspartate-β-hydroxylase (ASPH), a transmembrane protein widely expressed in cancer cells, in tumor progression and calcium homeostasis [22–23]. As a result, ASPH represents a relevant target for CaEP-based cancer therapies.Additionally, prolonged stress from calcium overload and membrane disruption may contribute to the generation of reactive oxygen species (ROS) [24–26]. Many cancer cells exhibit elevated ROS levels, which promote tumor progression, but they also maintain high antioxidant capacities to counteract oxidative stress. This delicate ROS balance plays a crucial role in cancer cell survival, and shifting it toward ROS-induced apoptosis could be a promising therapeutic strategy [27]. Targeting colorectal cancer cells with CaEP may help exploit their oxidative vulnerability, leading to selective tumor destruction.

Mitochondrial calcium overload and subsequent ROS production may also lead to oxidative damage in cellular structures such as the nucleus, potentially affecting the expression of death effector domain-containing DNA-binding protein (DEDD) [28, 29].

Furthermore, calcium influx and ROS generation are linked to heat shock protein (HSP) expression [30–32]. HSP plays the role of chaperons, typically producing in response to stressor signals such as heat, oxidative stress, inflammation, viral and bacterial infection, ischemia, or other physics factors. It protects cell molecules against denaturation and removes misfolded ones [33]. Notably, HSPs are frequently overexpressed in cancer cells, which play a role in apoptosis inhibition and immune modulation [34]. Necrotic or stressed cells may release HSPs into the extracellular environment, where they act as damage-associated molecular patterns (DAMPs) that stimulate both innate and adaptive immune responses [35, 36]. Investigating the role of HSPs in colorectal cancer cells following nsEP and CaEP exposure may provide further insights into their immunomodulatory potential.

Additionally, nsEP has been shown to influence immune system activation [37]. Understanding the impact of different CaEP protocols on HSP expression in cancer cells is essential for optimizing treatment strategies. Since electroporation affects lipid and protein organization in the cell membrane, it may also influence the expression of glycoproteins such as CD133, a cancer stem cell (CSCs) biomarker. CSCs exhibit unique redox mechanisms contributing to drug resistance, high treatment tolerance, and cell survival [38, 39]. Investigating the effects of CaEP on oxidative stress regulation in CSCs is critical to determining whether this approach can effectively target both tumor cells and the CSC population. Reducing CSC survival is particularly crucial in colorectal cancer, as these cells are often associated with tumor relapse and resistance to conventional therapies.This study focuses on the cellular responses to µsEP and nsEP in the presence and absence of calcium ions. We evaluate key factors related to cancer cell viability, energy metabolism, and calcium homeostasis, including ATP levels, ROS generation, and ASPH expression. Our goal is to assess the potential of µsEP and nsEP, combined with Ca²⁺, to trigger anticancer responses and inhibit cancer stem cell growth, particularly in colorectal cancer cells. By exploring these mechanisms, we aim to provide valuable insights into the therapeutic potential of CaEP as a strategy for enhancing cancer treatment efficacy.

## Materials and methods

### Cell culture

The three cell lines were tested – LoVo (sensitive human colorectal adenocarcinoma cell line), LoVo Dx (doxorubicin-resistant human colorectal adenocarcinoma cell line, Hs738st.int (normal human intestine fibroblast). The F-12 K Nut Mix medium (Nutrient Mixture Kaighn’s Modification, Gibco) for LoVo, LoVo Dx, and DMEM (Dulbecco’s Modified Eagle’s Medium, Sigma-Aldrich, St. Louis, MO, USA) for Hs738st.int were used, both supplemented with 10% FBS (Fetal Bovine Serum, Sigma-Aldrich, St. Louis, MO, USA), 1% antibiotic (streptomycin/penicillin, 10.000 µg/ml, 10.000 units/ml, Gibco). Cells were cultivated at 37 °C in a humidified atmosphere containing 5% CO_2_ and harvested or passaged by trypsin (0.025% trypsin and 0.02% EDTA; Sigma-Aldrich).

### Calcium electroporation protocols

Microsecond electric pulses were induced by the BTX 830 Electroporator (ECM830 Square Wave Electroporation System; BTX, Syngen Biotech, Wroclaw, Poland), and nanosecond electric pulses were generated by the PPG-20 generator (FID Technology, Germany). The 4 mm gap cuvettes (BTX, Syngen Biotech, Poland) were used for both procedures. Cells were suspended in 270 µl of HEPES buffer (10 mM HEPES (Lonza), 250 mM sucrose, and 1 mM MgCl_2_ in sterile water) with/without 30 µl of 20 mM CaCl_2_. Cells were divided into groups: untreated cells; positive control treated with 1 mM of staurosporine; cells treated with 2 mM of Ca^2+^; cells electroporated without Ca^2+^: 1.2 kV/cm ( 8 pulses, 1 Hz, 100 us), 37.5 kV/cm (200 pulses, 10 Hz, 10 ns) and 50 kV/cm (200 pulses, 10 Hz, 10 ns); cells electroporated with Ca^2+^: (2 mM of Ca^2+^ with 1.2 kV/cm, 37.5 kV/cm, 50 kV/cm). After the experiment, each group was incubated at 37 °C for 10 min, and the particular assays described in the next paragraphs were followed.

### MTS assay

After CaEP preformation, the cells were seeded in 96-well plates (3.1 × 10^4^ cells per 100 µl) in 6 repetitions of each parameter and incubated at 37 °C for 24 and 72 h. Subsequently, 20 µl of MTS agent (CellTiter 96^®^ AQueous One Solution Cell Proliferation Assay (MTS)) was added to each well and incubated for 2 h at 37 °C. Then, the absorbance was measured at 570 nm with the microplate reader (GloMax, Promega, Walldorf, Germany). The results were analyzed using a percentage of viable cells compared to untreated control cells.

### SRB assay

The SRB assay was performed after 72 h of incubation by introducing 50 µL of 50% trichloroacetic acid (TCA) into each well (incubation for 1 h at 4 °C). Afterward, it was rinsed five times with running water, left to dry entirely, and 50 µL of 0.4% sulforhodamine B (SRB) solution, prepared in 1% acetic acid, was added to each well (incubation for 30 min at RT). Excess dye was removed by washing the plate with 1% acetic acid, followed by drying. Next, 150 µL of 10 mM TRIS buffer was added to each well (incubation for 30 min at RT), and absorbance was then measured at 540 nm using a multi-plate reader (GloMax, Promega, Walldorf, Germany). The results were expressed as a percentage of viable cells relative to untreated controls.

### ROS level investigation

The ROS-Glo™ H₂O₂ Assay (Promega, G8820) is a sensitive bioluminescent assay that directly measures cell culture’s reactive oxygen species (ROS) level. Cells treated with CaEP protocol were seeded in a 96-well plate (80 µl with 20.000 cells/well) in 3 repetitions of each parameter, and 20 µl of H_2_O_2_ Substrate solution per well was added and incubated at 37 °C for 6 h (final concertation of H_2_O_2_ Substrate was 25 µM). Subsequently, the 50 µl of media from each sample well were removed to a 96-well white plate and mixed with 50 µl of ROS-Glo Detection Solution (prepared according to product protocol). After 20 min incubation at room temperature, the luminescence was measured with the multi-plate reader (GloMax, Promega, Walldorf, Germany).

### ATP level measurement

The ATP level was measured by The CellTiter-Glo^®^ Luminescent Assay (Promega, G7570), where mono-oxygenation of luciferin is catalyzed by luciferase in the presence of ATP. The luminescence signal intensity is proportional to the number of living cells and ATP level. The cells treated with CaEP protocols were seeded in a 96-well plate (100 µl with 50.000 cells/well) in 3 repetitions for each parameter. Subsequently, after 1, 2, 5, 24, and 48 h the 100 µl of CellTiter agent was added to each well for 10 min at room temperature, and the luminescence was measured with the microplate reader (GloMax, Promega, Walldorf, Germany).

### Immunofluorescence staining

The immunofluorescence staining technique analyzed three protein expressions – two heat shock proteins (HSP27, HSP70) and aspartate β-hydroxylase (ASPH). Cells treated with CaEP according to 2.2 Section were incubated on cover glasses in Petri dishes for 24 h and then fixed with 4% formaldehyde for 10 min. Samples were blocked with 4% BSA for 3 h, and the primary antibody anti-HSP27 (1:200 concentration, ab2790) and anti-HSP70 mouse monoclonal (1:200, Santa Cruz Biotechnology, sc-32239) were incubated at 37 °C for 3 h. Subsequently, primary antibodies were removed by washing three times in PBS, and the goat anti-mouse secondary antibody, Alexa Fluor 488 (1:500, Invitrogen, A-11029), was incubated at 37 °C for 1 h. Anti-ASPH mouse monoclonal antibody conjugated to Alexa Fluor 488 (Santa Cruz Biotechnology, sc-271391) was incubated at 37 °C for 1 h. Finally, each sample was washed thrice in PBS and mounted in FluorshieldTM with DAPI (4,6-diamidino-2-phenylindole) to stain nuclei. The images were performed using the Olympus FluoView FV1000 confocal laser scanning microscope (Olympus, Tokyo, Japan) with oil immersion objective lenses 60X.

### Immunocytochemistry procedures

The expression of the CD133 marker and Death Effector Domain-containing DNA binding protein (DEDD) were analyzed by immunocytochemistry staining. After 24 and 72 h, electroporated cells were fixed with 4% formaldehyde and stained according to the ABC Immunocytochemistry kit (Abcam, ab236466) procedure. The two antibodies were used: Recombinant Anti-CD133 antibody (1:200; Abcam, ab276130) and Anti-DEDD Antibody (1:100; Santa Cruz Biotechnology, sc-271192), both incubated overnight.

## Results

### Viability results

**Fig. 1:**
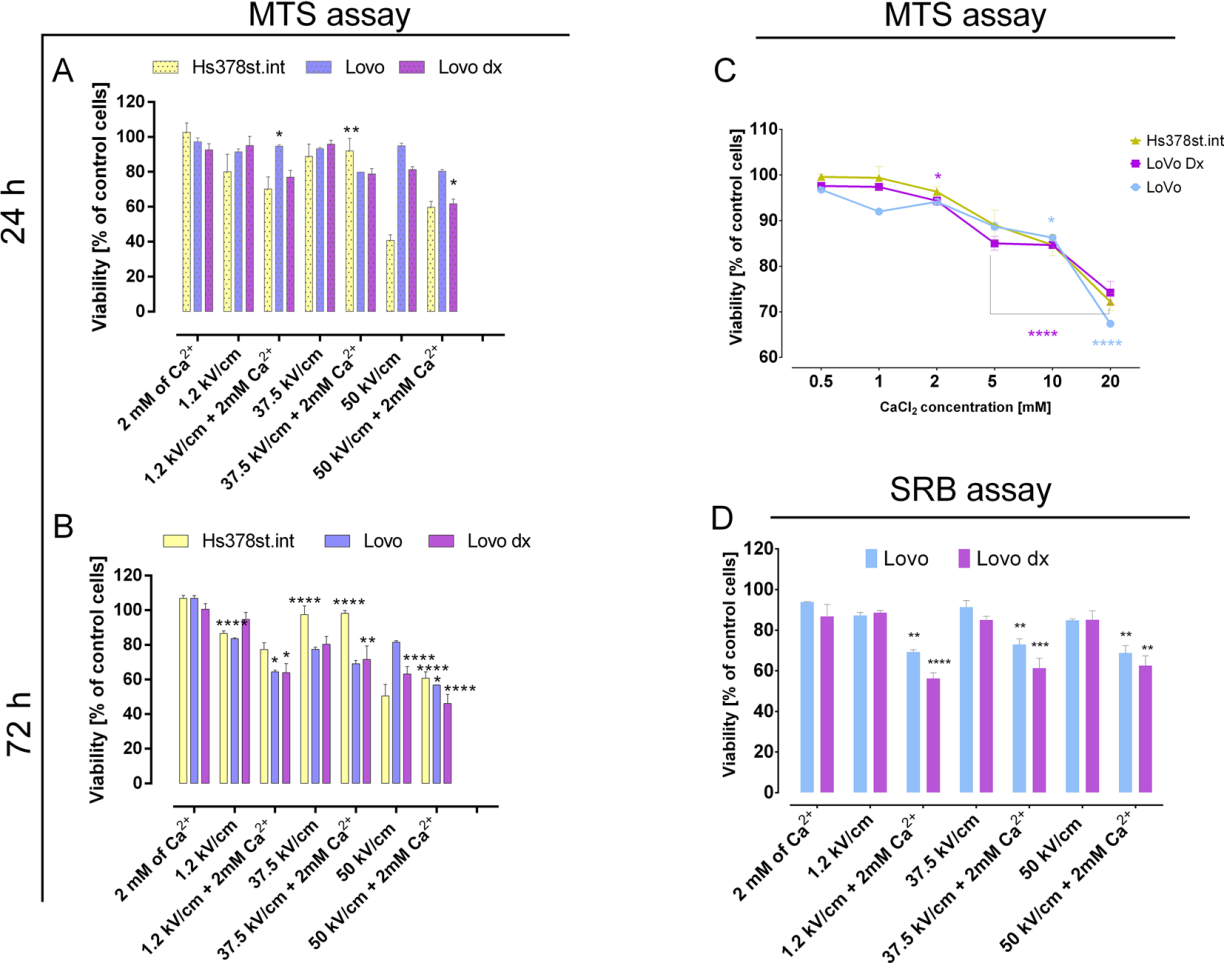
The effect of CaCl₂ and electroporation on cell viability assessed by MTT and SRB assays. (**A**) Cell viability of LoVo, LoVo Dx, and Hs378st.int after 24 h of treatment with electroporation (1.2 kV/cm, 8 pulses, 100 us, 1 Hz; 37.5 kV/cm and 50 kV/cm, 200 pulses, 10 ns, 10 Hz) with or without 2 mM Ca²⁺, measured by MTS assay. (**B**) The MTS assay assessed the cell viability of the same cell lines under the same conditions after 72 h. **(C**) Cell viability of LoVo, LoVo Dx, and Hs378st.int cell lines after 24-hour exposure to increasing concentrations of CaCl₂, assessed using the MTS assay. (**D**) The viability of LoVo and LoVo Dx cells after 72 h of treatment with electroporation (1.2 kV/cm, 8 pulses, 100 us, 1 Hz; 37.5 kV/cm and 50 kV/cm, 200 pulses, 10 ns, 10 Hz) with or without 2 mM Ca²⁺, evaluated using the SRB assay. Results are expressed as a percentage of control cell viability. Mean ± SD, *n* ≤ 6; *p < 0.05, **p < 0.01, ***p < 0.001, ***p < *0.0001*(comparison to control)

The MTS assay provides information about mitochondrion activity in cells exposed to various CaCl_2_ concentrations (Fig. 1C) and three different CaEP parameters (Fig. 1A, B). The results metric is the treated cells’ viability ratio compared to the untreated cells. Figure 1C illustrates the relationship between CaCl₂ (calcium chloride) concentration and cell viability (% relative to control). All treated cell lines (LoVo – light blue, LoVo Dx – purple, and Hs378st.int – yellow) show decreased viability as CaCl₂ concentration increases. A concentration of 2 mM was selected for further studies due to its low toxic effect on normal and cancerous cells. Figure 1A and B depict cell viability following exposure to electroporation under different conditions at 24 h and 72 h, respectively. A significant decline in viability was observed after 72 h. Microsecond CaEP (1.2 kV/cm + 2mM of Ca^2+^) caused a 36–37% viability decrease in each tested cell line. Similar results were obtained with the nanosecond parameter (50 kV/cm), which was lethal for both cancer cell lines, but the normal cells (Hs378st.int) preserved above 60% of viability. Adding Ca^2+^ to nsEP (50 kV/cm + 2 mM of Ca^2+^) reduced the cell survival ratio even to 46% for LoVo Dx. Here, the viability drop between the normal cell line (Hs378st.int) and the malignant cell lines (LoVo cells and LoVo Dx) was 4- and 5-fold, respectively. Moreover, CaEP application substantially impacted the resisted malignant cell line (LoVo Dx).

The SRB assay (Fig. 1D) was performed to validate the MTS assay. The SRB assay utilizes the properties of the dye Sulforhodamine B, which binds stoichiometrically to cellular proteins. This means that the amount of bound SRB is proportional to the protein content and, consequently, the number of cells. The SRB assay results after 72 h (Fig. 1D) show a similar trend to the MTS test, indicating a decrease in cell viability following electroporation and exposure to Ca²⁺, particularly at higher voltages. LoVo and LoVo Dx cells respond similarly; however, LoVo Dx appears to exhibit slightly more excellent resistance to the applied conditions.

### ROS and ATP level

**Fig. 2:**
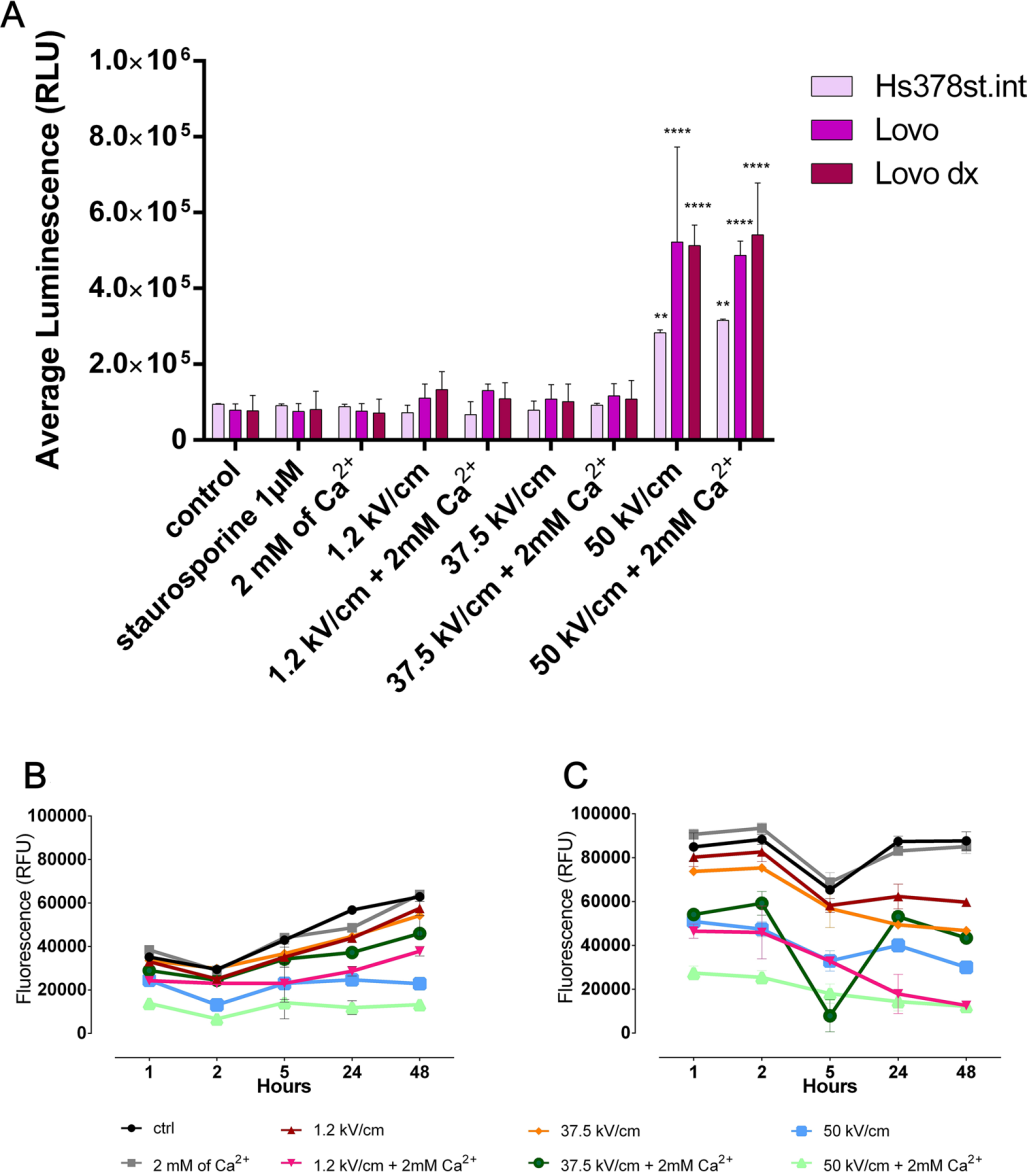
The reactive oxygen species (ROS) and ATP level measurement after EP +/- Ca^2+^ application in normal and malignant cell lines. (**A**) The ROS level was measured by ROS-Glo H_2_O_2_ assay 6 h after CaEP application. Hs378st.int (normal human intestine fibroblast), LoVo (sensitive human colorectal adenocarcinoma cell line), and LoVo Dx (doxorubicin-resistant human colorectal adenocarcinoma cell line) treated with 2 mM of calcium ions and electroporation (1.2 kV/cm, 8 pulses, 100 us, 1 Hz; 37.5 kV/cm and 50 kV/cm, 200 pulses 10 ns, 10 Hz). Results were presented as the average RLU with a standard deviation of duplicated samples. Mean ± SD; *N* ≤ 6; **p < 0.01, ****p < 0.0001, NS-not significant (comparison to control). The ATP level was measured by CellTiter-Glo Luminescence assay within 1 to 48 h timeframe after the EP protocols application: (**B**) LoVo (sensitive human colorectal adenocarcinoma cell line) and (**C**) LoVo Dx (doxorubicin-resistant human colorectal adenocarcinoma cell line) treated with 2 mM of calcium ions and electroporation (1.2 kV/cm, 8 pulses, 100 us, 1 Hz; 37.5 kV/cm and 50 kV/cm, 200 pulses 10 ns, 10 Hz). Results were presented as the average RLU with a standard deviation of duplicated samples. *N* ≤ 3; *p < 0.05, **p < 0.01, ***p < 0.001, ****p < 0.0001, NS-not significant (comparison to control)

Provided that the CaEP cytotoxic effect was confirmed, we examined the impact of CaEP on reactive oxygen species (ROS) generation, which are responsible for cell damage or death. Figure 2A shows the luminescence signal generated by luciferin, which is proportional to the ROS level in the tested sample. Only nsEP (50 kV/cm -/+ 2 mM Ca^2+^) elevated ROS level in all tested cell lines – for normal cell line ~2-fold and malignant cell lines 5-fold increase (compared to untreated cells). Malignant cell lines revealed a 3-fold higher ROS level than the normal cell line treated with the same parameters.

For better observability of the potential mitochondrial oxidative stress and energy depletion, we measured the ATP level after applying CaEP protocols (Fig. 2B-C). ATP level was monitored after 1,2,5,24, and 48 h against cancer cell lines. The three protocols caused significant ATP level decrease: µsCaEP (1.2 kV/cm + Ca^2+^) and nsEP (50 kV/cm +/- Ca^2+^). The energy reduction was more robust in LoVo Dx than in LoVo treated by the same protocols. It was correlated with the rise of ROS generation and a decline in viability. According to the literature, the disruption of ATP biosynthesis is linked with mitochondrial dysfunction and cell death. The following pattern was observed: to destabilize mitochondrion, µsEP needs to be supported by Ca^2+^, while nsEP alone is enough.

### ASPH expression

**Fig. 3:**
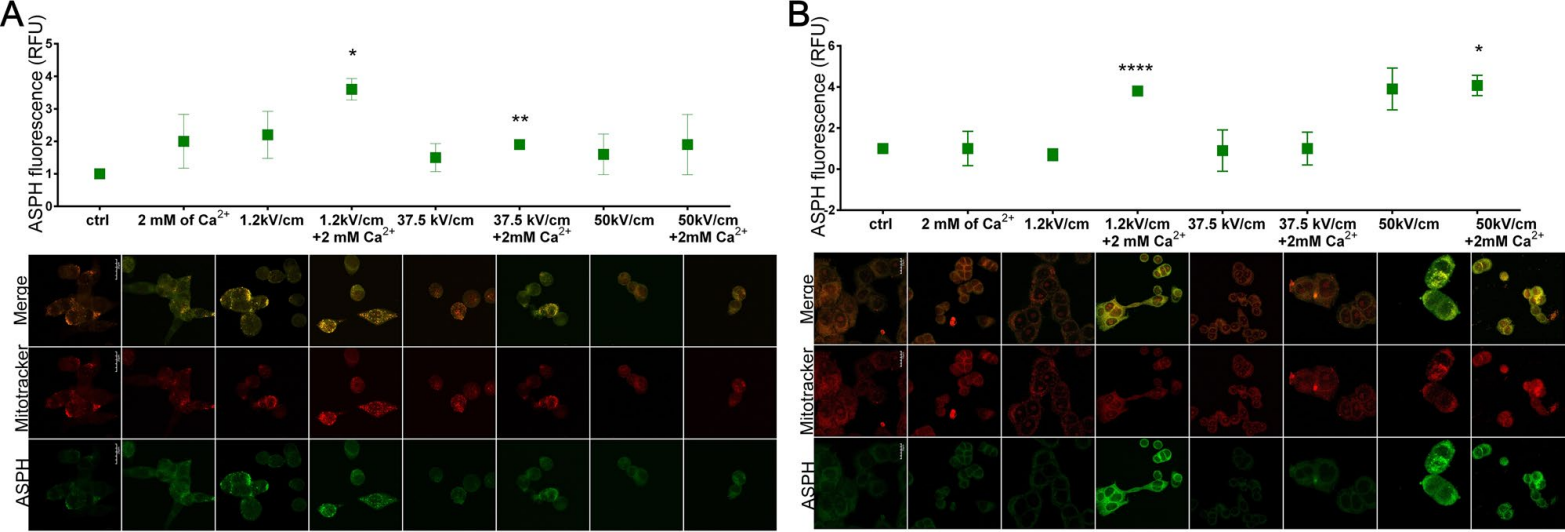
Immunofluorescence visualization of aspartate β-hydroxylase protein (ASPH) in malignant cell lines after EP +/- Ca 2 + application. (**A**) LoVo (sensitive human colorectal adenocarcinoma cell line) and (**B**) LoVo Dx (doxorubicin-resistant human colorectal adenocarcinoma cell line) treated with 2 mM of calcium ions and electroporation (1.2 kV/cm, 8 pulses, 100 us, 1 Hz; 37.5 kV/cm and 50 kV/cm, 200 pulses 10 ns, 10 Hz). Top panel: graphs present ASPH signal intensity for each tested therapy parameter; *n* = 4–5. Bottom panel: CLSM images of ASPH signal changes (green) and mitochondrion (red) visualized 72 h after EP protocols application; 20 μm; 60.0X oil immersion objective; NA: 1.35. *n* ≤ 3; *p < 0.05, **p < 0.01, ***p < 0.001, ****p < 0.0001, NS-not significant (comparison to control)

Immunofluorescence staining was used to evaluate the aspartate β-hydroxylase (ASPH) signal in treated cell lines (Fig. 3). ASPH is expressed by cancer cells; thus, the fluorescence signal was observed in both untreated cancer cell lines. The tested EP protocols evoked significant changes in ASPH signal in cancer cells. CaEP caused a radical increase of ASPH signal intensity in LoVo Dx compared to the control (1-fold and 1.5-fold for µsCaEP and nsEP 50 kV/cm +/- Ca^2+^, respectively). In LoVo, the fluorescence signal was lower than in LoVo Dx. Here, the expression of ASPH elevated after µsEP regardless of Ca^2+^ occurrence and nsCaEP (37.5 kV/cm + Ca^2+^). The normal cell line did not exhibit ASPH expression and only slightly increased the ASPH signal against the most robust CaEP parameter (50 kV/cm +/- 2 mM Ca^2+^). In LoVo Dx, the nsEP was enough to elevate the ASPH signal, while Ca2 + must support µsEP. Interestingly, the opposite pattern was observed in LoVo cells. Additionally, the co-localization with mitotracker red determined the strong association of ASPH with mitochondrion in both LoVo (PCC = 0.90 for 1.2 kV/cm; PCC = 0.74 for 1.2 kV/cm + Ca^2+^; PCC = 0.77 for 37.5 kV/cm + Ca^2+^) and LoVo Dx (PCC = 0.81 for 1.2 kV/cm + 2mM Ca^2+^; PCC = 0.68 for 50 kV/cm; PCC = 0.77 for 50 kV/cm + Ca^2+^). The untreated malignant cells exhibited a higher ASPH signal than the normal cell line, especially the doxorubicin-resistant human colorectal adenocarcinoma cell line, with a 1-fold increase of ASPH signal compared to normal cells (data not shown).

### HSP27/70 expression

**Fig. 4:**
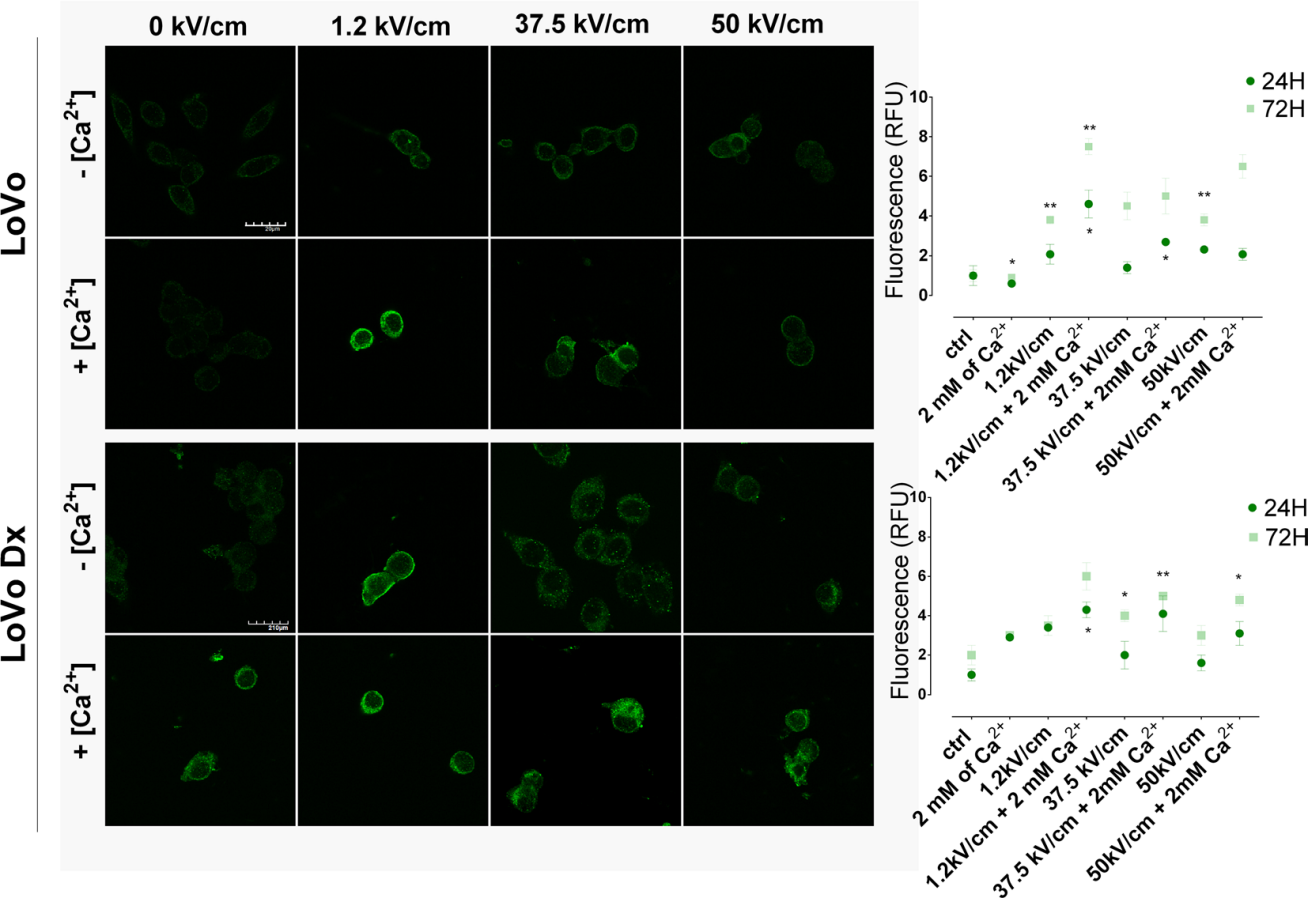
Immunofluorescence visualization of heat shock protein 27 (HSP27) in malignant cell lines after EP +/- Ca^2+^ application. LoVo (sensitive human colorectal adenocarcinoma cell line), and LoVo Dx (doxorubicin-resistant human colorectal adenocarcinoma cell line) treated with 2 mM of calcium ions and electroporation (1.2 kV/cm, 8 pulses, 100 us, 1 Hz; 37.5 kV/cm and 50 kV/cm, 200 pulses 10 ns, 10 Hz). Right panel: graphs present HSP27 signal intensity for each tested parameter 24 and 72 h after therapy; *n* = 4–5. Left panel: CLSM images of HSP27 signal changes (green) visualized 24 h after CaEP application; 20 μm; 60.0X oil immersion objective; NA: 1.35. *n* ≤ 3; *p < 0.05, **p < 0.01, ***p < 0.001, ****p < 0.0001, NS-not significant (comparison to control)

**Fig. 5:**
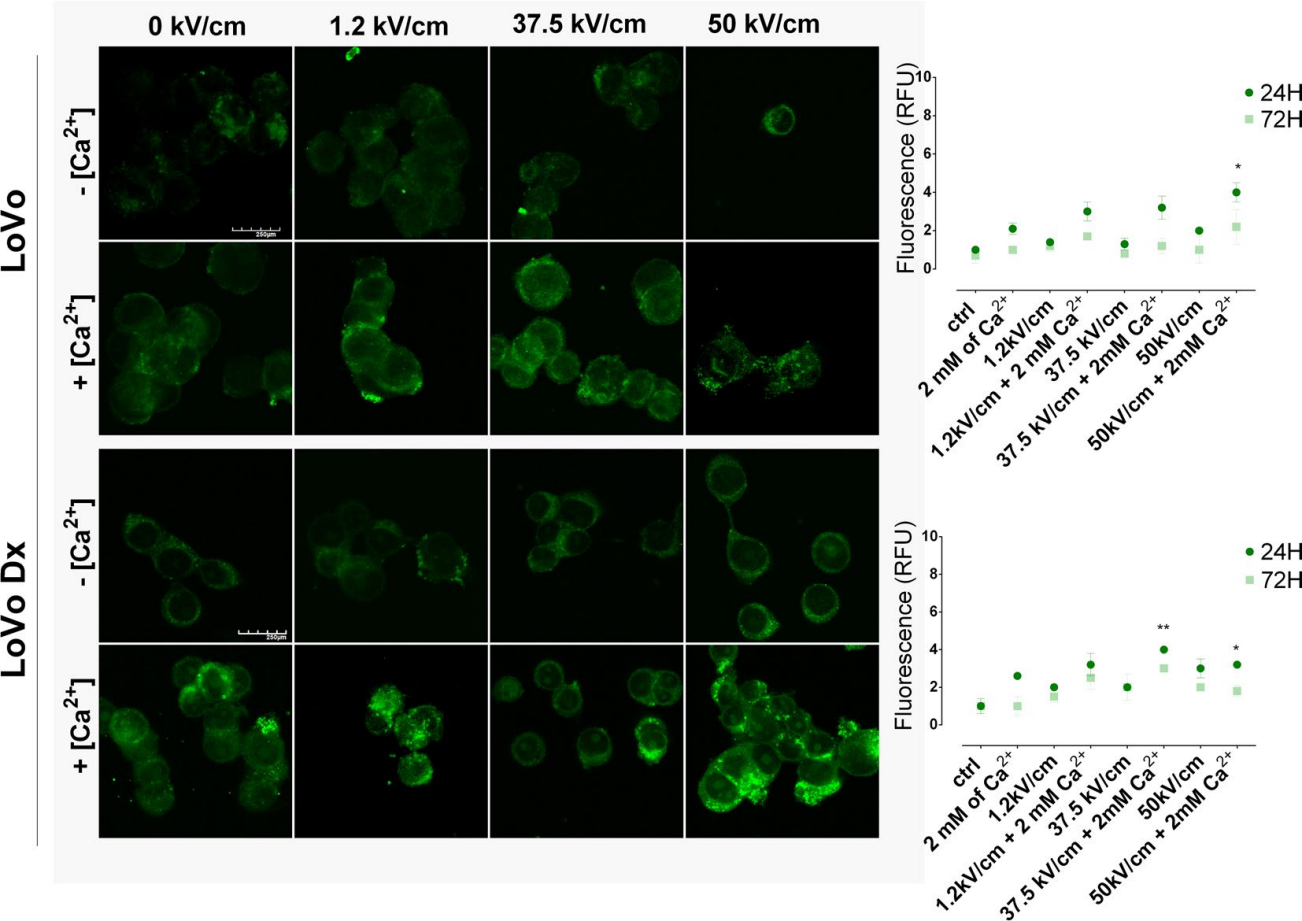
Immunofluorescence visualization of heat shock protein 70 (HSP70) in malignant cell lines after EP +/- Ca^2+^ application. LoVo (sensitive human colorectal adenocarcinoma cell line) and LoVo Dx (doxorubicin-resistant human colorectal adenocarcinoma cell line) treated with 2 mM of calcium ions and electroporation (1.2 kV/cm, 8 pulses, 100 us, 1 Hz; 37.5 kV/cm and 50 kV/cm, 200 pulses 10 ns, 10 Hz). Right panel: graphs present HSP70 signal intensity for each tested parameter 24 and 72 h after therapy; *n* = 4–5. Left panel: CLSM images of HSP70 signal changes (green) visualized 24 h after CaEP application; 250 μm; 60.0X oil immersion objective; NA: 1.35. *n* ≤ 3; *p < 0.05, **p < 0.01, ***p < 0.001, ****p < 0.0001, NS-not significant (comparison to control)

To indicate the impact of CaEP on the protection system and determine the difference between two tested cell lines, the following two heat shock protein levels were analyzed: heat shock protein 27 – HSP27 and heat shock protein 70 – HSP70. HSP belongs to the family of proteins, whose expression is triggered by stressful conditions. Figures 4 and 5 show the signal intensity of HSP27 and HSP70, respectively, 24 and 72 h after different parameters of CaEP application. The addition of Ca^2+^ during electroporation caused an increase in HSP70 fluorescence in both tested cell lines. HSP70 signal increased 2- and 3-fold after 24 h for µs- and nsEP with calcium in LoVo and LoVo Dx, respectively (compared to control cells). The same parameters as above triggered the elevation of HSP27 signal intensity in malignant cell lines: Lovo peaked after µsCaEP (3.6-fold for 1.2 kV/cm + 2mM Ca^2+^). At the same time, LoVo Dx exhibited vigorous signal intensity of HSP27 after µs- and nsCaEP, respectively. The measurement of the HSP fluorescence signal showed that the expression changed over time and that the HSP27 signal intensity was elevated in time in each tested parameter, whereas the HSP70 signal intensity was reduced. Fluorescence results are compatible with MTS tests and showed that the mortal therapy parameters triggered heat shock protein expression, and the survived cells activated protection mechanisms.

Similarly to the ROS-level assays, the heat shock protein level increased after applying the most cytotoxic parameters of nsEP + Ca^2+^. Both 27 and 70 HSP are involved in the anticancer immune response. The overexpression of HSP and its translocation to the tumor cell membrane is a strong stimulating signal for dendritic cells, which mediates the antitumor immune response.

### CD133 expression

**Fig. 6:**
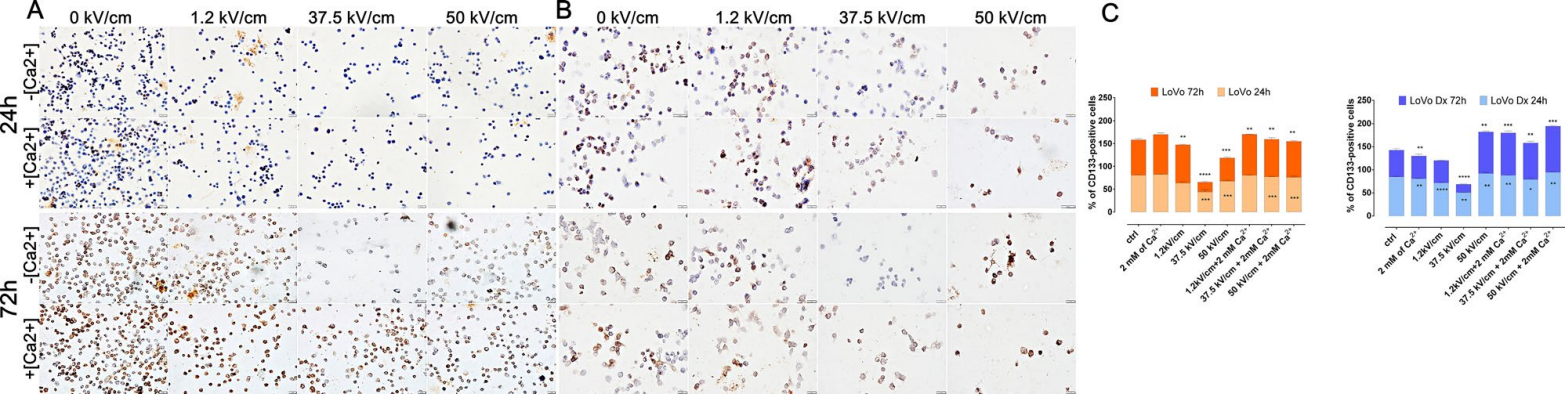
Immunocytochemistry analysis of CD133 marker expression in malignant cell lines in 24 h and 72 h after EP +/- Ca^2+^ application. (**A**) LoVo (sensitive human colorectal adenocarcinoma cell line) and (**B**) LoVo Dx (doxorubicin-resistant human colorectal adenocarcinoma cell line) treated with 2 mM of calcium ions and electroporation (1.2 kV/cm, 8 pulses, 100 us, 1 Hz; 37.5 kV/cm and 50 kV/cm, 200 pulses 10 ns, 10 Hz). Scale bar, 20 μm; (**C**) graphs present the percent of CD133-positive cells for each tested parameter 24 and 72 h after therapy. *n* ≤ 4; *p < 0.05, **p < 0.01, ***p < 0.001, ****p < 0.0001, NS-not significant (comparison to control)

The expression of CD133 was investigated in a 24–72 h window after the experiment (Fig. 6A-C). Both untreated, LoVo and LoVo Dx were CD133 positive with significant expression of the marker. The expression decreased after nsEP without Ca^2+^, and the parameter 37.5 kV/cm parameter provoked the most visible decline of CD133 expression in each tested cell line and timeframes. The calcium addition blocked the reduction of CD133 expression. Also, µsEP did not affect CD133 expression.

### DEDD expression

**Fig. 7:**
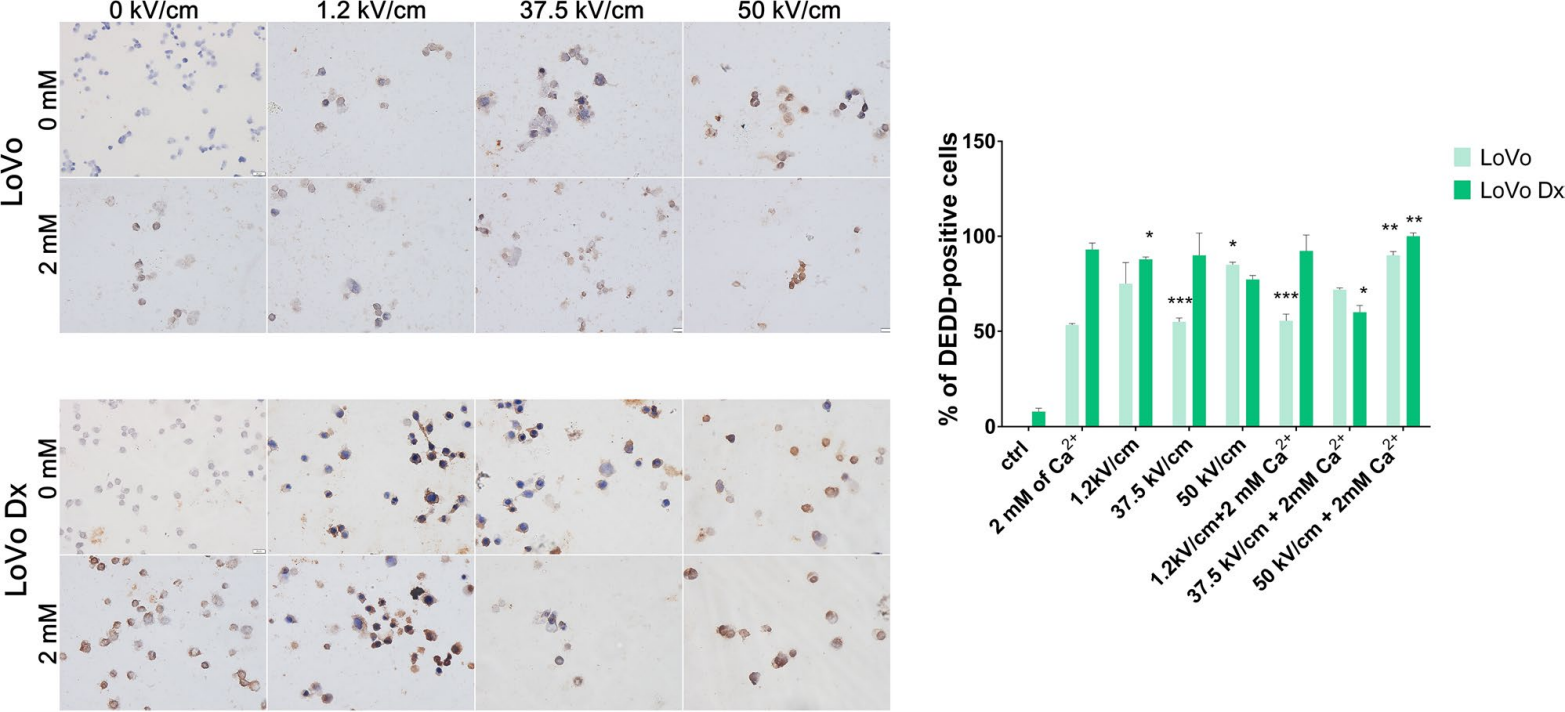
Immunocytochemistry analysis of DEDD protein expression in malignant cell lines in 72 h after EP +/- Ca^2+^ application. (**A**) LoVo (sensitive human colorectal adenocarcinoma cell line) and LoVo Dx (doxorubicin-resistant human colorectal adenocarcinoma cell line) treated with 2 mM of calcium ions and electroporation (1.2 kV/cm, 8 pulses, 100 us, 1 Hz; 37.5 kV/cm and 50 kV/cm, 200 pulses 10 ns, 10 Hz). Scale bar, 20 μm; (**B**) graph presents the percent of DEDD-positive cells for each tested parameter 72 h after therapy. *n* ≤ 3; *p < 0.05, **p < 0.01, ***p < 0.001, ****p < 0.0001, NS-not significant (comparison to control)

The death effector domain-containing DNA binding (DEDD) was visualized 72 h after the application of the protocols. As expected, The DEDD overexpression was inversely correlated with viability assays for cancer cell lines (Fig. 7A-B). Also, the addition of Ca^2+^ during electroporation has elevated the DEDD expression compared to electroporation alone. Electroporation (1.2 kV/cm and 37.5 kV/cm) caused the blebs containing DEDD occurrence. Interestingly, DEDD localization was different, in control and after usEP – cytoplasmic and after nsCaEP – nuclear. The most significant increase in DEDD expression was observed after nsCaEP (50 kV/cm + Ca^2+^).

## Discussion

Cancer cells have precise machinery that protects them from uncontrolled proliferation and stimulates invasiveness.

The study’s main goal was to compare nano- and microsecond calcium electroporation for sensitive and drug-resistance cancer cell lines (LoVo, LoVo Dx) and normal colon cell lines (Hs378st in), which has not been done yet.

The previous study showed that nano- and microsecond CaEP efficiently reduced cancer cell survival. The cytotoxicity effect of CaEP was proven in various cancer types [40–60], but the cellular responses triggered by different parameters of CaEP are still under investigation.

In our study, both µsCaEP and nsCaEP effectively reduced the viability of cancer cell lines, with a significantly more substantial impact on cancer cells than normal cells. Moreover, we found that calcium electroporation, especially nanosecond electroporation, triggered the increase of death effector domain – DEDD expression, mainly responsible for cell death pathway activation and cell cycle [61]. Interestingly, the localization of DEDD depends on the electroporation type, and the study showed that nuclear translocation of DEDD correlated with the reduction of cellular bioactivity by activating caspase-6 and RNA polymerase I inhibition. It has been reflected in viability results, which exhibited nuclear localization of DEDD and drastic cell viability after nsCaEP. According to the literature, DEDD triggers apoptosis, whereas CaEP activates a spectrum of cell death types like apoptosis, necrosis, necroptosis, or pyroptosis. Thus, deeper analyses are required to conform cell death pathway [62, 63].

EPOSE is well-established as an anticancer therapy and is widely used in clinical oncology, as well as in humans and veterinary. Higher efficiency of EP combined with various drugs (bleomycin, cisplatin) or compounds like calcium ions for cancer cell lines compared to normal cell lines has already been repeatedly proven [5, 10, 43, 44, 45, 58, 64–65]. Although, the differences in cell response depended on protocol application and cell lines. We observed various cell reactions in LoVo and LoVo Dx cell lines.

Many studies have proven the disparate permeabilization profile of different EP protocols [66–68]. Since the nsEP also permeabilizes intracellular organelles, crucial differences may be found in mitochondrion function [69–71]. µsEP causes the uptake of external Ca^2+^ whereas nsEP triggers also release of intracellular Ca^2+^ from ER-storages [42, 72]. Rapid Ca^2+^ influx overloads mitochondrion and activates various biochemical cascades [73–75]. Our study confirms that the ATP decreases mostly after nsEP, corresponding to mitochondrion disruption. nsEP, in contrast to µsEP, elevated the reactive oxygen species generation. Pakhomova et al. proved that nsEP could induce both intra- and extracellular ROS [76], whereas Novickij et al. obtained the same conclusion for EP with calcium ions [77]. Interestingly, Novickij et al. also demonstrated a strong generation of ROS by µsCaEP for lung cancer cells, which contradicts our results for colon cancer cells. Disbalance of Ca^2+^ and ROS homeostasis are common determinants of cell death. Oxidative stress and reactive oxygen species induce damage in the mitochondrion.

Maintenance of Ca^2+^ balance is engaged in numerous systems of channels, pumps, and proteins [78]. One is aspartate-β-hydroxylase (ASPH) – membrane glycoprotein located mainly in ER. ASPH is widely expressed in cancer cells and responsible for cancer proliferation, migration, and invasion [22, 79]. Recently, the expression of ASPH in mitochondrial fraction was proven to correlate with loss of mtDNA integrity and mitochondrial disruption [80]. Therefore, we submitted that the expression of ASPH after calcium electroporation might be changed. Our study has proven that overexpression of ASPH is correlated with mitochondrion disturbance triggered by nsEP or CaEP. The co-localization of ASPH with mitochondrion is demonstrable. Simultaneously, the link between viability decreases, ROS elevation, and mitochondrion disruption becomes increasingly natural.

Currently, the anticancer immune response has become highly challenging. Provocation of the immune system using electroporation is still very new, and only a few reports are showing an increase in pro-inflammatory cytokines level (IL-1β, IL-10, IL-6, TNF-α, IFNγ) in vivo and release of high mobility group box 1 (HMGB1) in vitro [81, 82]. Moreover, IRE also stimulates the immune system by IFNγ, calreticulin, HMGB1 release, and CD^8+^ T cells and dendritic cells [83–85]. Nowadays, the study indicates the crucial role of HSPs in anticancer immune response [36, 86, 87]. Stressors such as ROS, calcium overloading, and membrane perturbation activate prevention mechanisms. The heat shock proteins represent the highly conserved chaperons that allow cells to survive in stressful or lethal conditions [88, 89]. They are overexpressed in inflammation, hypoxia, or infections. Interestingly, HSP27 and 70 significantly increase after oxidative stress or cytotoxic drugs. The nsEP protocol is going to have tremendous potential to trigger HSP overexpression and translocation to the cell membrane, where they are potent stimulators for immune system components, such as dendritic cells [35, 37, 90]. We can conclude that µs- and nsEP + Ca^2+^ are cytotoxic for colon cancer cells (20–40% death cells). However, more importantly, these protocols overexpressed HSP27 and HSP70; thus, activation of immune response in LoVo and LoVo Dx by calcium electroporation is realistic and needs further study.

Colorectal cancer is rich in cancer stem cells (CSCs) or tumor-initiating cells (TIC) – a subpopulation of cells expected to embryonic stem cells, which lost control of proliferation and differentiation, leading to tumorigenesis. CSC divide asymmetrically to generate daughter and cancer cells, which provide rapid tumor growth and abnormal cell turnover, leading to therapy resistance [91, 92]. CSC/TIC produces specialized cancer cells, which interact with the immune system during immunoediting. Those clone cells modified genetically may produce new immunogenic peptides for immune cell elimination. Moreover, one of the less immunogenic CSC subpopulations evades the immune system and may promote slow tumor growth. Antigen-presenting molecules like MHC-I, MHC-II, co-inhibitory molecules (CTLA4), or co-stimulatory molecules (CD80, CD86) are crucial for presenting cancer antigens to T cells and triggering an immune reaction. CSC/TIC has shown downregulation of MHC-I and MHC-II expression [93]. It has been found that CSC/TIC secretes more cytokines such as TGF-β (transforming growth factor beta; in breast cancer and glioblastoma) or IL-4 (in colon cancer) than cancer cells reducing the immune response and escalating drug resistance [94, 95]. CSC/TIC generates anti-apoptotic molecules such as BCL-2 or BCL-xL, which protect them against chemotherapy and apoptosis.

Interestingly, CSC/TIC uses the STAT3 pathway for immunosuppression by blocking macrophage activity, reducing cytotoxicity of NK cells (natural killer), or even reducing expression of MHC molecules on dendritic cells due to impaired lymphocyte activation and antitumor immunity [96]. Therapies focused on CSC elimination are highly desirable for blocking tumor growth and invasiveness. The ideas like antibody constructs or vaccines have been developed. We used the CD133 marker for CSC labeling out of the many stem cell markers. CD133 is the transmembrane glycoprotein found in the normal brain, liver, prostate, kidney, skin, and cancer stem cells derived from the brain, lung, colon, ovary, skin, and pancreas. CD133 role is not precisely known, although a study demonstrated its involvement in tumor chemoresistance, tumorigenesis, and metastasis [97]. Studies showed the crucial role of CD133 in apoptosis prevention by high expression of FLIP – caspase 8 inhibitor and interaction with the PI3K-Akt pathway leading to activation of pro-apoptotic factors (MCL-1, BCL-2, BCL-XL). Cells with a high level of CD133 expression exhibited an increased proliferation ratio and reduced autophagy and apoptosis evens compared to cells with low CD133 expression. The CSCs are regarded as a high drug-resistance subpopulation of cancer cells, which can cause recurrence. CSCs exhibit resistance to standard chemo- and radiotherapy [98]. The specific redox status of CSCs and maintenance of the reactive oxygen species on the optimal level seem to underlie the preservation of self-renewal and tumor-recovery capabilities. CSCs produce high levels of antioxidants and exhibit reduced concentrations of ROS (superoxide) compared to cancer cells or even normal epithelial cells [99]. Our results are promising in reducing CSC viability by nsEP (especially by 37.5 kV/cm). We noticed a significant decrease in CD133-positive cells after nsEP treatment, but there is no evidence of direct associations with other processes investigated in this study.

Interestingly, the addition of Ca^2+^ during electroporation preserved up-regulated CD133 expression. That correlated with a high level of ROS and a decrease in ATP production. Griguer et al. proved that CD133 expression is not mandatory for stem cells, but overexpression is induced in human glioma cells by mitochondrion dysfunction or hypoxia [100]. Additionally, the renal CD133 + progenitor cells (scattered tubular cells (STCs)) were able to repair ischemic kidney injury. The CD133 + STCs transferred the mitochondrion to injured tubular cells via tunneling nanostructures [101]. The CD133 glycoprotein seems closely related to mitochondrion functions in normal and cancer stem cells. Since CaEP strongly impacts the mitochondrion, causing bioenergetic stress, the role of cells CD133 + might be vital for cancer cells’ survival, and further analysis is demanding.

In conclusion, calcium electroporation (CaEP) with microsecond and nanosecond pulses effectively reduced cancer cell survival, especially in sensitive and drug-resistant cancer cell lines. The study showed that CaEP triggered an increase in death effector domain (DEDD) expression, which is responsible for cell death pathway activation and cell cycle regulation. The permeabilization profile of different electroporation protocols causes different cell reactions and mitochondrial functions. Aspartate-ß-hydroxylase (ASPH) overexpression is correlated with mitochondrial disruption triggered by nanosecond electroporation or CaEP. Electroporation has the potential to stimulate the immune system response and the overexpression of heat shock proteins (HSPs), which play a crucial role in the anticancer immune response. The nsEP impacts the decrease of the CD133 marker, which seems essential to the tumor formation mechanism, self-renewal capacity, and drug resistance. Further analyses are required to confirm the cellular mechanisms of calcium electroporation and the potential of various electroporation protocols in clinical oncology.

## Conclusion

Calcium electroporation (CaEP) using microsecond and nanosecond pulses effectively reduces the survival of cancer cells, particularly drug-resistant types, by increasing the expression of the death effector domain (DEDD), which regulates cell death pathways. Different electroporation protocols affect cell reactions and mitochondrial functions, with nanosecond pulses disrupting mitochondria and increasing reactive oxygen species (ROS). CaEP also stimulates immune responses by overexpressing heat shock proteins (HSPs). Notably, nanosecond pulses decrease the CD133 marker, associated with tumor growth and drug resistance. Further research is needed to explore CaEP’s full potential and mechanisms in cancer therapy.

## Data Availability

The datasets used and/or analysed during the current study are available from the corresponding author on reasonable request.

## Abbreviations

ASPH: Aspartate-β-hydroxylase
ATP: Adenosine triphosphate
BCL-2: B-cell lymphoma 2
BCL-xL: B-cell lymphoma-extra large
CaEP: Calcium electroporation
CICR: Calcium-induced calcium release
CLSM: Confocal laser scanning microscope
CSC: Cancer stem cells
CTLA4: Cytotoxic T-lymphocyte-associated protein 4
DAMP: Damage-associated molecular pattern
DEDD: Death Effector Domain Containing
ECT: Electrochemotherapy
EP: Electroporation
ER: Endoplasmic reticulum
FLIP: Fas-associated death domain-like interleukin-1-β-converting enzyme-inhibitory protein
IL-1β: Interleukin-1β
IL-10: Interleukin-10
IL-6: Interleukin-6
IFNγ: Interferon gamma
HMGB: High mobility group box
HSPs: Heat shock proteins
MHC: Major histocompatibility complex
MCL-1: Induced Myeloid Leukaemia Cell Differentiation Protein
NK: Natural killer
PI3K/Akt: The phosphatidylinositol-3-kinase/Akt signaling pathway
µsEP: Microsecond electroporation
nsEP: Nanosecond electroporation
nsCaEP: Nanosecond calcium electroporation
STAT3: Signal transducer and activator of transcription
STCs: Scattered tubular cells
ROS: Reactive oxygen species
TIC: Tumor-initiating cells
TGF-β: Transforming growth factor beta
TNF-α: Tumor Necrosis Factor
